# Association Between Wearable Device Use and Levels of Physical Activity Among Older Adults in the US: Evidence From the 2019-2020 Health Information National Trends Survey

**DOI:** 10.7759/cureus.44289

**Published:** 2023-08-28

**Authors:** Anita O Onyekwere, Okelue E Okobi, Francis C Ifiora, Micheal K Akinboro, Ngozi T Akueme, Joy Iroro, Abigail O Dan-Eleberi, Faith C Onyeaka, Aba Amoasiwah Ghansah

**Affiliations:** 1 School of Medicine, Richmond Gabriel University, Kingstown, VCT; 2 Family Medicine, Larkin Community Hospital Palm Springs Campus, Miami, USA; 3 Family Medicine, Medficient Health Systems, Laurel, USA; 4 Family Medicine, Lakeside Medical Center, Belle Glade, USA; 5 Pharmacy, University of Texas Health Science Center at Houston, Houston, USA; 6 Epidemiology and Biostatistics, Texas A&M Health School of Public Health, College Station, USA; 7 Dermatology, University of Medical Sciences (UNIMED), Ondo, NGA; 8 Internal Medicine, All Saints University School of Medicine, Roseau, DMA; 9 Internal Medicine, University of Ghana Medical School, Accra, GHA; 10 Haematology/Blood Transfusion Science, Madonna University, Calabar, NGA; 11 Internal Medicine, Kwame Nkrumah University of Science and Technology, Accra, GHA

**Keywords:** activity, elderly, us, usa, older adults, physical activity, wearable device, association

## Abstract

Objective

To examine the relationship between electronic wearable device (WD) use and physical activity (PA) levels among older adults in the US.

Methods

Data were pooled from 3310 older adults from the 2019 and 2020 Health Information National Trends Survey. The explanatory variable was WD use, and the outcomes were weekly PA levels, resistance training, and sedentary time. Logistic regression was conducted to investigate the association between WD use and the reported outcome variables. Separate logistic models were also fitted to explore the relationship between WD use and physical activity outcomes among a subgroup of older adults with chronic conditions.

Results

A total of 14.4% of older adults reported WD use. Older adults who use WD were more likely to meet national guidelines for weekly levels of PA (odds ratio (OR) 1.60, 95% confidence intervals (CI) (1.10, 2.32); p = 0.015) and resistance strength training (OR 1.54, 95% CI (1.14, 2.09); p = 0.005) when compared with their counterparts not using WD. After restricting the analysis to those with chronic conditions only, WD use was only associated with a higher level of weekly strength training (OR 1.68, 95% CI 1.19, 2.38; p = 0.004).

Conclusion

WD use may be associated with increased physical activity among older adults, including those with chronic health conditions. Further studies are needed to examine the factors influencing the adoption and sustained use of WD in older adults.

## Introduction

Today, 15.2% of the U.S. population is over the age of 65, growing by over a third (34.2%) in the last decade [1]. This proportion is expected to double by 2030 and further aggravate the current imbalance of healthcare provider demand and supply caused by health worker shortages across the country [1,2]. Advancements in early disease detection and therapeutics have largely contributed to people living longer [3]. Yet, older adults have elevated rates of chronic diseases and higher use of healthcare systems. Consequently, Medicare spending accounted for 21% of total national healthcare expenditures in 2019 [4]. To address these challenges, measures that prevent the occurrence of chronic diseases among ageing populations through lifestyle modifications need to be encouraged.

Physical activity (PA) is an important lifestyle factor that has been demonstrated to reduce the risk of developing chronic diseases such as coronary heart disease, stroke, type 2 diabetes mellitus, obesity, and hypertension [5-7]. Alongside the prevention of chronic diseases, PA has also been shown to improve mental health, decrease cognitive decline, and reduce overall mortality in older adults [8-10]. The Federal Physical Activity Guidelines for Americans [11] recommend 150 minutes per week of moderate-level PA for adults 65 years of age and older. Despite empirical evidence supporting the health benefits of PA, research and national survey estimates have consistently reported suboptimal levels of physical activity in older adults in the United States [12,13]. Thus, it is essential that innovative interventions be implemented to promote PA among older adults.

Wearable devices (WD) have become increasingly popular in the last decade. WDs are electronic devices attached to watches and clothing worn by a consumer that can continuously capture biometric information related to health or fitness. WD tracks daily activities such as sleep, heart rate, physical activity, and mobility patterns [14]. It has been shown to improve physical activity levels by sending motivational reminders to users, which allows them to compare their physical activity levels with their peers and evaluate the direct impact of their actions on physiological parameters like blood pressure [15,16]. Furthermore, data from some WD can be integrated into health data systems, which can assist clinicians in capturing patient data better [17]. Despite these reported benefits of WD, only 17% of adults aged 65 years or older in the US are WD users [18].

Past research has examined the use of WD for promoting PA among older adults in the US [19-21]. One study [21] examined the feasibility, acceptability, and effect of WD and telephone counseling on the physical activity levels of 40 older adults and concluded that WD was feasible, acceptable, and resulted in increased levels of physical activity among older adults. Another study of WD usage [20] involving 22 rural-dwelling older adults also reported increased levels of physical activity. Although results from these studies showed the benefit of increased PA among WD users, these studies were limited by small sample sizes and restricted to subpopulations, and as such, were not nationally representative. Additionally, a few systematic reviews have examined the link between WD and PA in older adults and found modest increases in PA among WD users. However, these reviews were limited by methodological constraints, including the heterogeneity of studies, the diverse age range of study participants, and the small number of randomized trials [22,23].

Accordingly, in this study, we used a nationally representative sample to (i) examine rates of ownership of WD and (ii) investigate the association between WD use and physical activity parameters among older adults in the United States. As wearable technologies have become more prevalent, it is relevant to evaluate how older adults engage with wearable technology to manage their physical health due to the potential for WD to positively impact physical activity levels among older adults. The information gained from this study will provide population-level evidence that may help guide physical activity intervention plans for older adults.

## Materials and methods

Procedures

For this study, we extracted data from the fifth iteration of the Health Information National Trends Survey (HINTS), which is a nationally representative household interview survey of civilian noninstitutionalized U.S. adults aged ≥18 years that has been administered by the National Cancer Institute (NCI) every few years since 2003. Although the HINTS primarily collects information about cancer diagnosis, treatment, and survivorship, it also collects information on several other topics from the general population, including lifestyle behaviors and the utilization of health communication systems between providers and patients. The data used in this study is from cycles three and four of the fifth iteration of the HINTS, which were collected from January 22 to April 30, 2019, and February 24 to June 15, 2020, respectively.

Complete information about data collection and methodologies, including the sampling and weighting processes of both HINTS datasets, has been described in previous publications [24]. Briefly, both iterations of HINTS 5 employed a two-stage, stratified random sampling technique. The first stage involves the selection of non-vacant residential addresses obtained from the Marketing Systems Group (MSG). In the second stage, an adult from each household was selected for participation in the survey using the ‘Next Birthday’ method. The database of residential addresses was grouped into two categories: high-minority strata (areas with ≥34% Hispanics or African Americans) and low-minority strata (areas with <34% Hispanics or African Americans). This stratification was done to increase the precision of estimates for minority subpopulations. The survey respondents were then weighted to reflect selection probabilities and to provide a nationally representative sample in terms of age, gender, educational attainment, marital status, race, ethnicity, and census region. In addition to the full-sample weight, a set of 50 replicate weights was provided for each adult. These replicate weights are used to calculate the standard error of estimates obtained from the HINTS data using the delete one jackknife (JK1) replication method [25]. Written informed consent was obtained from study participants. HINTS 5 cycles 3 and 4 were approved by the Westat Institutional Review Board and classified as exempt from review by the US National Institutes of Health Office of Human Subjects Research Protections because the data were de-identified.

Ethical approval and public data use

The HINTS system stands out as a publicly accessible resource, offering an array of information for both public health professionals and the general populace. This user-friendly platform provides convenient access to extensive data stores. The profound insights derived from this survey play a pivotal role in facilitating well-informed decision-making and fostering a more profound comprehension of health trends and disparities among the non-institutionalized civilian population in the United States. Importantly, the publicly accessible website [11,24,26] offers access to data and information that fall within the public domain. Such data can be employed, duplicated, shared, or published without necessitating explicit permission or undergoing an Institutional Review Board (IRB) review. This leniency stems from the fact that the analysis revolves around de-identified data and does not qualify as human subject research (HSR), as outlined in 45 CFR 46.102.

Study design and participants

A cross-sectional design was used to evaluate participant responses from both HINTS 5 cycle 3 (H5C3) and HINTS 5 cycle 4 (H5C4). The combined H5C3 and H5C4 datasets contained responses from 9,303 survey respondents: 5,438 and 3,865 respondents from cycles 3 and 4, respectively. Our inclusion criteria were based on (i) respondent age >/= 65 years and (ii) response to the survey question "In the last 12 months, have you used an electronic wearable device to monitor or track your health or activity?". Participants who did not respond to this question (or those for whom data was "missing") were excluded from the analyses. We categorized the survey respondents into two groups based on their responses to the survey question in (ii): wearable device users and wearable device non-users.

The overall household response rate was 30.3% in H5C3 and 37% in H5C4. Of note, participants surveyed at each cycle were different people, and consequently, this was not a repeated measures design. Of the complete sample, we identified a total of 3,370 adults ≥ 65 years. Of these, 3,310 responded to the sentinel survey question on electronic wearable device use and thus were included in the analyses. About 14.4% of the study participants (496/3,310) were wearable device users.

Measures

Ownership and Patterns of Wearable Device Use

The main exposure variable was usage or ownership of WD within the past 12 months preceding the survey. This was determined by the following questions: "In the past 12 months, have you used an electronic wearable device to monitor or track your health or activity? For example, a Fitbit, Apple Watch, or Garmin Vivofit"? Next, willingness to share health information from a WD was ascertained from the survey question: Would you be willing to share health data from your wearable device with your healthcare provider? Response options were "yes" or "no".

Finally, the frequency of WD utilization was derived from the survey question: In the past month, how often did you use a wearable device to track your health? Response options ranged from "every day", "almost every day", "one to two times per week", "less than once per week" and "I did not use a wearable device in the past month". For this study, given the small number of affirmative responses to the frequency of WD utilization question, we reclassified the frequency of WD as never users, infrequent users (one to two times per week or less), and frequent users (>2 times per week).

Physical Activity Parameters

We studied three physical activity-related outcomes: weekly physical activity, strength training, and sedentary behavior. All three outcomes were ascertained using questions derived from the HINTS survey. Specifically, physical activity was determined using the following two survey questions: (i) In a typical week, how many days do you do any physical activity or exercise of at least moderate intensity, such as brisk walking, bicycling at a regular pace, and swimming at a regular pace? Response options ranged from none to seven days per week, and (ii) on the days that you do any physical activity or exercise of at least moderate intensity, how long do you do these activities? Response options ranged from minutes to hours. Responses to these two questions (the days of physical activity and the time of each day) were multiplied to obtain the weekly average time for physical activities for each respondent. Next, based on the Federal Physical Activity Guidelines for Americans and World Health Organization (WHO) recommendations and guidelines for physical activity levels [11,26], we reclassified the respondents into two groups for physical activity levels, that is, whether the subject (i) met the physical activity recommendations (≥150 minutes per week) or did not meet the physical activity recommendations (<150 minutes per week). Next, the level of daily sedentary behavior was assessed from the survey question: During the past seven days, how much time did you spend sitting on a typical day at home or at work? Response options ranged from 0 to 20 hours. Based on previous literature on recommendations for sedentary behavior [27], sedentary behavior was classified as ‘high’ if the respondent spent eight or more hours a day sitting and as ‘low’ if the respondent spent less than eight hours a day sitting.

Finally, the weekly level of strength training was determined from the survey question: In a typical week, outside of your job or work around the house, how many days do you do leisure-time physical activities specifically designed to strengthen your muscles? (for e.g., weightlifting, resistance band exercises, and bodyweight exercises), Response options ranged from one day per week to seven days per week. Strength training was dichotomized based on national recommendations for resistance and strength exercise of ≥2 times/week into inadequate (<2 times/week) and adequate (≥2 times/week).

Covariates

Covariates included in the present study were gender, race, marital status, educational level, household income, insurance status, comorbidities, access to a regular healthcare provider, confidence in one's own ability to take care of one's health, and self-health status. Respondents were classified as having a comorbidity if they had one or more of the following conditions: diabetes mellitus, hypertension, heart disease, lung disease, cancer, or depression. Self-health status was ascertained from the survey question, "In general, would you say your health is excellent, very good, good, fair, or poor?’ This was recoded into three categories: "very good or excellent", "good," and "fair or poor" health. Our inclusion of variables was informed by previous studies [28].

Statistical analyses

We first conducted basic descriptive statistics for the entire study sample and by WD status. Weighted percentages were presented. Chi-squared tests were used to compare WD use across respondents' sociodemographic and health-related characteristics. Next, three separate multivariable logistic regression models were estimated to investigate the relationship between WD use and physical activity parameters (weekly physical activity, daily sedentary behavior, and weekly strength/exercise resistance training) in the entire study population, adjusting for gender, race, marital status, educational level, household income, insurance status, comorbidities, access to a regular health care provider, confidence in one's own ability to take care of one's health, and self-health status. Next, we performed a subgroup analysis and ran further sets of multivariable models adjusting for the same covariates to examine the association of WD use among a subset of older adults with chronic conditions. Then we performed further exploratory analysis using multivariable logistic regression models to evaluate the association between frequency of WD use and all three physical activity parameters, adjusting for the same covariates.

We performed all statistical analyses using the "svy" command in Stata 17.0 statistical software (StataCorp LP, College Station, Texas, USA). Final person weights and jack-knife replicate weights provided within the H5C3 and H5C4 datasets were used to estimate national-level values and standard errors of estimates, respectively. All tests were two-sided, and p values < 0.05 were considered statistically significant.

## Results

We identified a total of 3,370 adults ≥ 65 years. Of these, 3,310 responded to the sentinel survey question and were included in the analyses. As depicted in Table 1, overall, 54% of the population were women, 78% were white, 50% had less than college-level education, and over 80% reported at least a good self-health status. A total of 498 participants (weighted percentage: 14.4%) endorsed the past 12-month use of WD. Among the older adults using WD, a substantial proportion (395/498, 76.9%) indicated their willingness to share health data from their WD with their healthcare providers. There was no significant difference in WD usage by gender (53.9% vs. 46.1%, p-value = 0.062). However, insured individuals (14.65% vs. 0.39%, p <0.0001) and those from households with an annual income of at least $75,000 were more likely to use WD, and Hispanics were the least likely of all the racial groups to use WD. Full details on the distribution of the sociodemographic characteristics of the study population by WD use are shown in Table 1 below.

**Table 1:** Sample population demographic characteristics

Demographic variables	Total (n=3310), %	Wearable device non-users (n=2812), %	Wearable device users (n=498), %	p-value
Gender	-	-	-	0.062
Female	53.87	84.14	15.86	-
Male	46.13	87.45	12.55	-
Age group	-	-	-	<0.001
65-69	32.74	79.40	20.60	-
70-74	25.64	85.51	14.49	-
75-79	17.68	87.24	12.76	-
80+	23.94	93.07	6.93	-
Marital status	-	-	-	0.014
Not married	41.68	87.97	12.03	-
Married	58.32	83.93	16.07	-
Education	-	-	-	<0.001
Less than college	50.13	89.53	10.47	-
Some college	27.99	85.49	14.51	-
College graduate	10.77	79.84	20.16	-
Post-graduate	11.11	73.35	26.65	-
Household income	-	-	-	<0.001
< $20,000	19.05	93.49	6.51	-
$20,000 - $34,999	17.46	92.35	7.65	-
$35,000 - $49,999	16.40	91.37	8.63	-
$50,000 – $74,999	20.11	83.40	16.60	-
$75,000 or more	26.98	73.19	26.81	-
Race	-	-	-	0.035
White	78.03	84.61	15.39	-
African American	9.21	82.88	17.12	-
Hispanic	8.43	92.89	7.11	-
Others	4.33	81.01	18.99	-
Insurance status	-	-	-	<0.001
Not insured	0.85	99.61	0.39	-
Insured	99.15	85.35	14.65	-
Self-health status	-	-	-	<0.001
Fair or poor	19.34	91.59	8.41	-
Good	39.81	88.82	11.18	-
Excellent or very good	40.85	79.70	20.30	-
Comorbidities	-	-	-	0.632
None	16.37	84.54	15.46	-
Yes	83.63	85.84	14.16	-
Confidence in taking care of own health	-	-	-	0.082
Somewhat or none	29.36	88.04	11.96	-
Completely or very	70.64	84.60	15.40	-
Having a regular provider	-	-	-	0.141
No	18.32	88.98	11.02	-
Yes	81.68	84.91	15.09	-
Strength training	-	-	-	<0.001
Inadequate (<2 times/week)	71.26	88.18	11.82	-
Adequate (>/= 2 times/week)	28.74	78.54	21.46	-
Physical activity	-	-	-	<0.001
Inactive (< 150mins/week)	67.84	88.94	11.06	-
Active (>= 150mins/week)	32.16	78.49	21.51	-
Sedentary levels	-	-	-	0.1135
Low levels (<8 hours/day)	65.02	84.15	15.85	-
High levels (>/= 8 hours/day)	34.98	86.69	13.31	-

WD and physical activity

Older adults who used WD were more likely to achieve nationally recommended weekly levels of physical activity (odds ratio (OR) 1.60, 95% confidence intervals (CI) (1.10, 2.32); p = 0.015) and resistance strength training (OR 1.54, 95% CI (1.14, 2.09); p = 0.005) when compared with their counterparts not using WD after adjusting for differences in sex, age category, educational level, insurance status, marital status, race, income level, access to a regular healthcare provider, confidence in their own ability to take care of their own health, self-health, and presence of comorbidities. There was, however, no statistically significant difference in meeting past seven-day daily sedentary levels between users and non-users of WD (OR 0.97, 95% CI (0.68, 1.38); p = 0.876). Results from both the unadjusted and fully adjusted models are shown in Table 2 below.

**Table 2:** Multivariable logistic regression for the association between WD and PA Levels among older adults *Adjusted for sex, age category, educational level, insurance status, marital status, race, income level, health care provider status, confidence in own ability to take care of health, self-health, and presence of comorbidities. WD: wearable device; PA: physical activity; OR: odds ratio; CI: confidence intervals

Outcomes	Unadjusted OR (95% C.I.)	p-value	Adjusted OR (95% C.I.)	p-value
Meet weekly physical activity (>= 150mins/week)	2.20 (1.64, 2.96)	<0.001	1.60 (1.10, 2.32)	0.015
Meet weekly strength training (>= 2days/week)	2.04 (1.58, 2.62)	<0.001	1.54 (1.14, 2.09)	0.005
Daily sedentary behavior (> 8 hours/day)	0.82 (0.63, 1.05)	0.116	0.97 (0.68, 1.38)	0.876

In an additional exploratory analysis, we evaluated the association between the frequency of WD usage and similar physical activity outcomes. After adjusting for the same sociodemographic and health-related factors, we found that compared to never users of WD, frequent users of WD are more likely to achieve nationally recommended weekly levels of physical activity (OR 2.05, 95% CI 1.35, 3.09; p = 0.001) and resistance strength training (OR 1.75, 95% CI 1.25, 2.45; p = 0.001). There was, however, no statistically significant difference in the odds of achieving nationally recommended physical activity levels (OR 0.92, 95% CI 0.49, 1.71; p = 0.786) or resistance strength training (OR 1.21, 95% CI 0.64, 2.28; p = 0.556) between never-users and infrequent users of WD. Complete details on both the unadjusted and fully adjusted models are shown in Table 3 below.

**Table 3:** Multivariable Logistic regression for the frequency of WD use and physical activity levels among older adults in the US The primary predictor for each model is the frequency of wearable device use (never user, infrequent user, or frequent user; never user is the reference category). Models were *adjusted for sex, age category, educational level, insurance status, marital status, race, income level, health care provider status, confidence in one's own ability to take care of one's own health, self-health, and presence of comorbidities. WD: wearable device; PA: physical activity; OR: odds ratio; CI: confidence intervals

Variables	Unadjusted OR OR (95% C.I.)	p-value	Adjusted OR^*^ OR (95% C.I.)	p-value
Outcome: weekly PA level ("reference group" is "never user")				
Infrequent user	1.32 (0.76, 2.31)	0.320	0.92 (0.49, 1.71)	0.786
Frequent user	2.87 (2.09, 3.98)	<0.001	2.05 (1.35, 3.09)	0.001
Outcome: strength training (reference category for primary predictor is ‘never user’)				
Infrequent user	1.39 (0.89, 2.17)	0.144	1.21 (0.64, 2.48)	0.556
Frequent user	2.45 (1.78, 3.38)	<0.001	1.75 (1.25, 2.45)	0.001
Outcome: sedentary Level (reference category for primary predictor is ‘never user’)				
Infrequent user	1.26 (0.76, 2.07)	0.363	1.28 (0.66, 1.71)	0.467
Frequent user	0.64 (0.47, 0.88)	0.006	0.83 (0.54, 1.27)	0.390

Lastly, we assessed the relationship between WD and physical activity among older adults with chronic diseases (N = 2824). After adjusting for the same set of covariates, WD users were more likely to achieve nationally recommended weekly levels of resistance strength training (OR 1.68, 95% CI 1.19, 2.38; p = 0.004) than non-users. There was, however, no statistically significant difference in achieving recommended daily sedentary levels (OR 0.99, 95% CI (0.69, 1.43); p = 0.955) or weekly physical activity levels (OR 1.44, 95% CI (0.97, 2.12); p = 0.069) between users and non-users of WD among older adults with chronic diseases. Results from both the unadjusted and fully adjusted models are shown in Table 4 below.

**Table 4:** Multivariable logistic regression of association between WD with physical activity levels among older adults with chronic diseases (n = 2824) *Adjusted for sex, age category, educational level, insurance status, marital status, race, income level, health care provider status, confidence in own ability to take care of health, self-health, and presence of comorbidities. WD: wearable device; OR: odds ratio; CI: confidence intervals

Outcomes	Unadjusted OR (95% C.I.)	p-value	Adjusted OR (95% C.I.)	p-value
Meet weekly physical activity (>= 150mins/week)	2.33 (1.72, 3.14)	<0.001	1.44 (0.97, 2.12)	0.069
Meet weekly strength training (>= 2days/week)	2.24 (1.68, 2.99)	<0.001	1.68 (1.19, 2.38)	0.004
Daily sedentary behavior (> 8 hours/day)	0.79 (0.59, 1.06)	0.116	0.99 (0.69, 1.43)	0.955

## Discussion

This paper sought to examine how the use of WD may be associated with engagement in physical activity among older adults in the US. Specifically, we investigated the prevalence of WD adoption, the willingness to share data from WD with clinicians, and the association between the use of WD and general physical activity, resistance exercise, and sedentary behaviors among older adults, drawing from a large nationally representative sample of older adults in the US. To gain further insights into the clinical utility of WD in chronic disease management, we also explored the relationships between WD use and physical activity engagement among older adults with chronic diseases.

Findings suggest that among older adults, adoption of WD remains low. We found that only one in seven (14.4%) of older adults reported using WD in the 12 months preceding the survey. Our finding of 14% WD ownership among older adults is lower than estimates of 21% to 28% reported in the general adult population from multiple recent national assessments [18,28-29]. Yet, the low adoption rates of WD among older adults in our study sample align with estimates (8% to 12.5%) reported in other older adult populations [30-32]. While there is some evidence to support the increasing uptake and popularity of digital technology among older adults nationally [33,34], it is also possible that barriers unique to older adults may account for their lower rates of WD adoption relative to the general population.

Previous studies have reported the perceived usefulness of technological tools, confidence in using technology, design factors, digital literacy, negative attitudes, and age-related decline in cognitive, sensory, and motor function as significant challenges to digital tool adoption among older adults in the literature [35-38]. As the technology landscape continues to evolve and wearable devices become more popular for health management, it is critical to develop technology tools that are readily accessible and usable by older populations.

Nonetheless, one encouraging finding from our analysis is that among older adults who endorsed using WD, a substantial proportion are willing to share their wearable health data with healthcare providers. Our analysis demonstrated that of the older adults who endorsed WD ownership, about 65% reported daily or almost daily use of these devices, and 76.9% reported willingness to share data from their WD with their health providers. These results corroborate findings from past works indicating similarly high rates of willingness to share healthcare data with clinicians, researchers, or public health agencies among users of WD [18,29,39]. Since a substantial proportion of older adults are willing to share their wearable health data with their healthcare providers, WD provides a valuable opportunity for clinicians to continuously engage, monitor, collect data, and support positive health behaviors to enhance health outcomes in this highly vulnerable group [19,32,40,41].

Electronic WD also represents a new opportunity to support behavioral change and promote physical activity in older adult populations. After adjusting for sociodemographic and health-related factors, we found that older adults who used WD were 1.54 times more likely to achieve nationally recommended weekly levels of physical activity and 1.60 times more likely to meet the national recommendation for weekly strength training than those who reported not using WD. These findings are in line with results from multiple cross-sectional studies, systematic reviews, and meta-analyses on the association between WD and physical activity [28,42-44]. However, we found no significant statistical difference between the use of WD and daily sedentary activity among older adults. Thus, although WDs have the potential to facilitate positive health behavior change, our data indicates that these devices alone may not be sufficient to drive behavioral change. While the wearable health market continues to exponentially grow and represents a feasible tool for positive behavioral lifestyle change, our finding of a lack of association between WD use and sedentary activity in older adults suggests that the efficacy of these tools in promoting behavioral change remains to be proven [45,46].

The intensity of WD use may play a role in promoting positive health changes. Previous works examining the role of wearable healthcare devices in facilitating physical activity have mainly focused on the association between ever-users vs. non-users of WD and physical activity engagement [28,44]. These studies indicated that simply using wearable devices may promote physical activity among adults. In our analysis, we found that compared to nonusers of WD, older adults who reported frequent past-month use of WD (daily or almost daily use) were more likely to achieve nationally recommended weekly levels of physical activity and strength training than their counterparts who reported not using WD in the past month. We found no differences between older adults who endorsed infrequent use of WD over the past month and non-users of WD with respect to physical activity engagement. Our findings extend the literature and suggest that the frequent and sustained use of WD may be associated with improved physical activity engagement. This finding is supported by research indicating that frequent use of digital health tools may potentiate any positive health benefits derived from their use through habit [47,48]. Despite their popularity, interest in WD may wane with time, resulting in suboptimal long-term adherence [49], which may undermine any initial early benefits attained from their use. Our results provide early evidence regarding the intensity of WD use and positive lifestyle change and support the notion that active use, not simply owning WD, may lead to better health outcomes.

Wearable devices are clinically useful and can positively impact the physical health of adults with chronic diseases [50]. In our subgroup analysis restricted to older adults with medical comorbidities (diabetes, hypertension, cancer, lung disease, heart disease, and depression), we found that WD was associated with increased physical activity (meeting the weekly recommendation for resistance strength training) among older adults with chronic medical conditions. Our results suggest the potential to utilize WD as a tool to stimulate physical activity in chronically ill older adult populations. Thus, although studies indicate that complications from chronic diseases such as vision impairment, cognitive deficits, and mobility issues may limit older adults' use of digital health tools [38,51], WD may have the potential to promote physical activity among older adults with chronic medical conditions. Therefore, further research is needed to evaluate strategies aimed at improving the adoption of WD in older adult populations to ensure the proper integration of these tools in their care.

Strengths and limitations

Strengths of this study include the use of HINTS, a large dataset, which ensures that our study is sufficiently powered to detect any differences. Also, the use of survey weights enables the generalization of the study results to the US older adult population.

Several limitations are worth mentioning. First, the cross-sectional nature of the data limits our ability to draw causal conclusions between WD use and physical activity measures. We could not also report on the impact of WD use on PA behaviors over time. Second, the use of secondary data limits our ability to examine other theoretically relevant factors such as the reasons for wearable device use, the duration of WD use, and the specific type of WD. Third, the use of self-reported PA measures could induce some recall and social desirability bias. Although self-reported PA measures are often less accurate and prone to bias when compared to device-based PA measurements [52,53,54], they remain a cost-effective means of collecting PA data for population surveillance and continue to be utilized in multiple public health epidemiological studies [55]. Therefore, future research should consider employing robust longitudinal designs and randomized trials to better comprehend the intersections and temporal order between wearable technologies and physical activity. Fourth, the potential for reverse causality in explaining our findings cannot be ruled out. It is possible that older adults with higher levels of PA may be more likely to use WD for their PA. Lastly, the low response rates in both HINTS iterations have the potential to introduce selection bias. However, our use of sampling weights could have mitigated the low response rate, and the fact that the overall weighted prevalence of WD was still low and in keeping with other data suggests our sample was representative and not prone to selection bias issues.

## Conclusions

Our study provides useful insights into the state of WD utilization among older adults in the US. We highlight that only 14.4% of older adults owned WD, and of these, about 76.9% reported a willingness to share data from their WD with their health providers. We also found that the use of WD was associated with positive physical activity. From a clinical perspective, our results indicate that WD may serve as a potential tool to achieve the recommended level of physical activity among older adults, including those with some chronic conditions. These findings suggest that WD may have positive physical health benefits in older populations and underscore the need to improve the usage of WD among this population. Thus, future studies should examine the factors influencing the adoption and sustained use of WD in older adult populations.
